# Influence of Inter-Training Intervals on Intermanual Transfer Effects in Upper-Limb Prosthesis Training: A Randomized Pre-Posttest Study

**DOI:** 10.1371/journal.pone.0128747

**Published:** 2015-06-15

**Authors:** Sietske Romkema, Raoul M. Bongers, Corry K. van der Sluis

**Affiliations:** 1 University of Groningen, University Medical Center Groningen Department of Rehabilitation Medicine, Groningen, the Netherlands; 2 University of Groningen, University Medical Center Groningen, Center of Human Movement Sciences, Groningen, the Netherlands; MRC Institute of Hearing Research, UNITED KINGDOM

## Abstract

Improvement in prosthetic training using intermanual transfer (the transfer of motor skills from the trained, “unaffected” hand to the untrained, “affected” hand) has been shown in previous studies. The aim of this study is to determine the influence of the inter-training interval on the magnitude of the intermanual transfer effects. This was done using a mechanistic, randomized, single-blinded pretest-posttest design. Sixty-four able-bodied, right-handed participants were randomly assigned to the Short and Long Interval Training Groups and the Short and Long Interval Control Groups. The Short and Long Interval Training Groups used a prosthesis simulator in their training program. The Short and Long Interval Control Groups executed a sham training program, that is, a dummy training program in which the same muscles were trained as with the prosthesis simulator. The Short Interval Training Group and the Short Interval Control Groups trained on consecutive days, while the Long Interval Training Group and Long Interval Control Group trained twice a week. To determine the improvement in skills, a test was administered before, immediately after, and at two points in time after the training. Training was performed with the “unaffected” arm; tests were performed with the “affected” arm. The outcome measurements were: the movement time (the time from the beginning of the movement until completion of the task); the duration of maximum hand opening, (the opening of the prosthetic hand while grasping an object); and the grip-force control (the error from the required grip-force during a tracking task). Intermanual transfer was found in movement times, but not in hand opening or grip-force control. The length of the inter-training interval did not affect the magnitude of intermanual transfer effects. No difference in the intermanual transfer effect in upper-limb prosthesis training was found for training on a daily basis as compared to training twice a week.

**Trial Registration:**

Nederlands Trial Register NTR3888

## Introduction

Intermanual transfer means that a motor skill trained in one arm will also improve that skill in the other arm [1–4], and can therefore be used to improve prosthetic training [5–7]. When applying intermanual transfer after an upper-limb amputation, the unaffected arm can be trained in prosthetic skills by using a prosthesis simulator, which is operated in the same way as an actual prosthesis. As a result, prosthetic training can start even before the wounds are healed and the prosthesis is obtained. Starting to train within the first four weeks after amputation is important, because it leads to better prosthetic handling and acceptance [8–10].

Intermanual transfer effects, after training with an upper-limb prosthesis simulator, have been demonstrated previously [5–7]. In our earlier studies training sessions took place on five consecutive days [6,7], which differs from most other articles on intermanual transfer where training took place within one session [4,5,11–15]. Interestingly, in literature on training not only did the number of sessions but also the duration between sessions, that is, inter-training intervals, affect training [16–18]. In between training sessions, the motor skill becomes consolidated, which would seem to be taking place especially during sleep [19,20]. The time necessary for the consolidation, and thus the length of the inter-training interval, depends on diverse aspects of the task, such as its complexity [17]. Although such an effect from the inter-training interval has been demonstrated only once in intermanual transfer [21], it has been shown that learning improves with inter-training intervals lengthened up to 24 hours [16,17].

Studies concerning inter-training intervals only occasionally measure intervals longer than 24 hours [18,22–25]. In the scant studies focusing on inter-training intervals in motor learning, intervals that are lengthened from days to months do not seem to further improve learning [24,25]. More to the point, a decrease in learning is actually seen for longer intervals, which is also observed in verbal-recall tasks [18,22]. This suggests that there is an optimal inter-training interval, in which the consolidation of learning is highest [17,22,26,27].

The aim of this study is to compare the effects of two inter-training intervals on intermanual transfer in upper-limb prosthetic training. Therefore, the presence of intermanual transfer and whether this effect differs between inter-training intervals has been tested by comparing the Short and Long Interval Training Groups with the Short and Long Interval Control Groups receiving a sham training program. The sham training program consisted of a dummy training (playing Nintendo Wii and playing darts) without using the simulator where the coordination of comparable wrist muscles was trained. We hypothesize that the inter-training interval of 24 hours will have a larger transfer effect than the longer inter-training interval.

## Materials and Methods

The protocol for this trial and supporting CONSORT checklist are available as supporting information; see S1 Checklist and S1 Protocol.

### Participants

**Fig 1:**
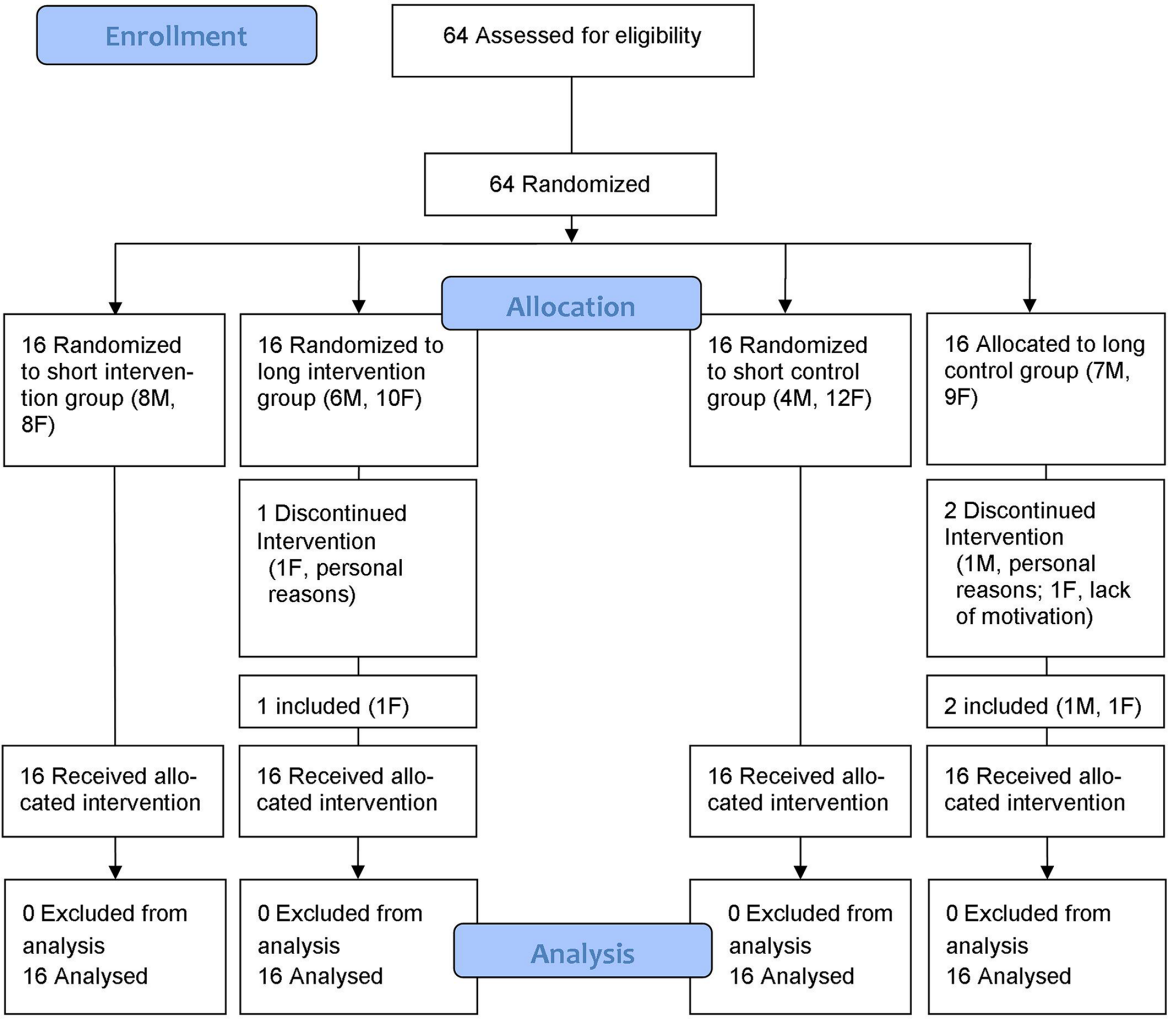
CONSORT flow diagram.

Sixty-four right-handed, non-amputated volunteers (Fig 1) were recruited from among university students, and were assessed between September and December 2013. Three participants left the training soon after inclusion due to lack of motivation. These participants were replaced by three participants of the same sex. All participants were free of known neurological or upper extremity musculoskeletal problems, had normal sight, and had no previous experience with the prosthesis simulator. Hand dominance was determined by the Edinburgh Handedness Inventory [28].

The sample size calculation was based on the data of an experiment in which participants also trained for five consecutive days with the prosthesis simulator. These participants executed the same grip-force control training and test tasks as in this study. A power analysis using Cohen's d was performed to determine the sample size. Using G*Power [29], we estimated that we needed 17 participants per group to reach a power of 0.8. A t-test with two independent means with an effect size of 0.89 and type I error of 0.05 was used. Controlling for an equal distribution of sex and test hand per group, and taking into account that in this study the training sessions are longer, we included 16 participants per group.

### Ethics Statement

All participants signed an informed consent document before participation. The study was approved by the local ethics committee (UMCG Medical Ethics Review Committee, NL43335.042.13). The trial was registered with the Nederlands Trial Register (NTR3888). After completion of the experiment, the participants received a gift voucher.

### Design overview

**Fig 2:**
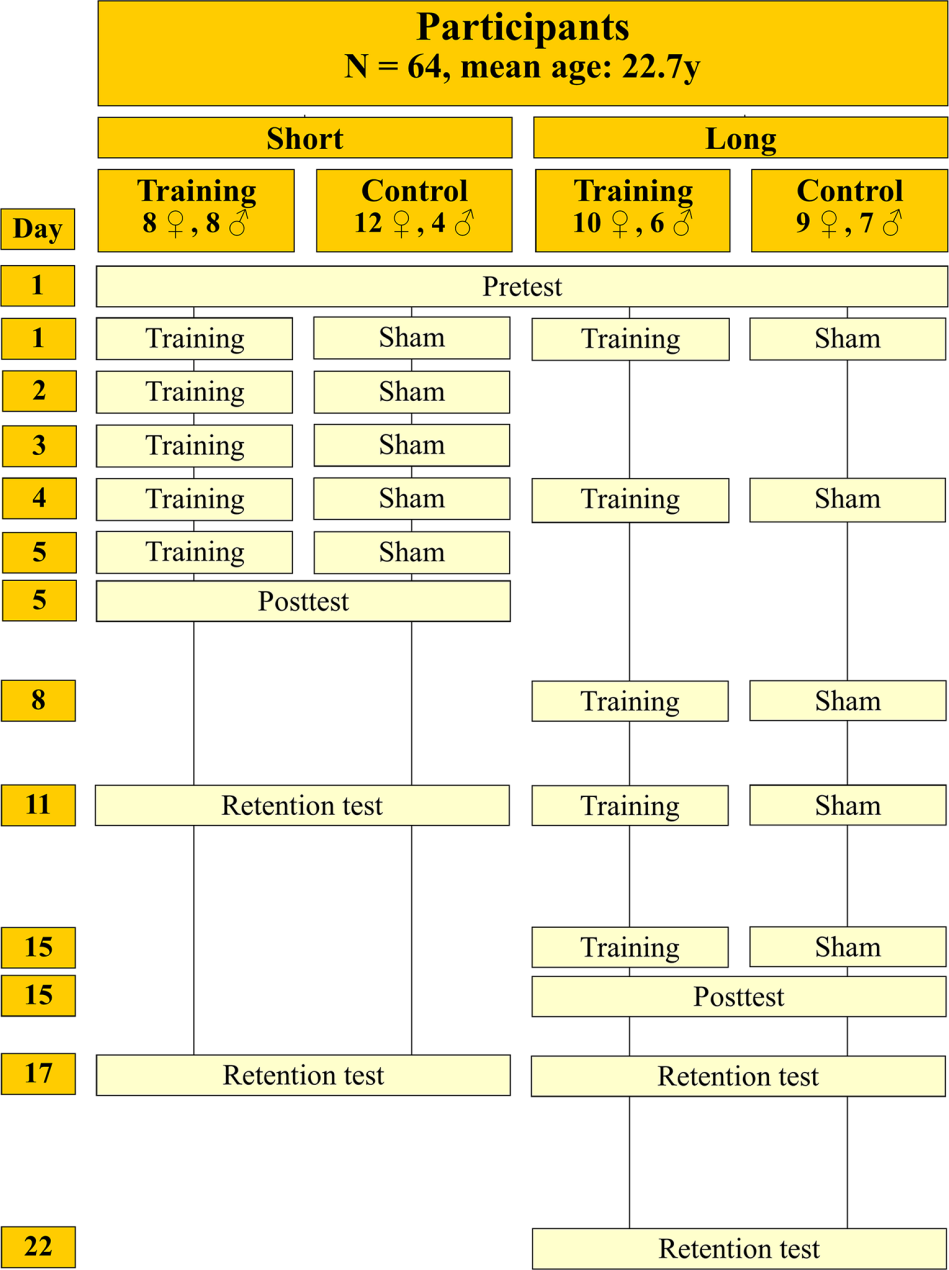
Design of the experiment.

Using a computer-generated random number sequence, the participants were pseudo-randomly assigned to one of four groups (Fig 2): two Training Groups that underwent a training program with the prosthesis simulator (the Short Interval Group with a short inter-training interval [24 hours] and the Long Interval Group with a long inter-training interval [>24 hours]), and two Control Groups that underwent a sham training program with the same intervals (the Short and Long Interval Group). The hand (preferred/right versus non-preferred/left side) that was used as the test hand was equally divided over the groups. This was done using a pseudo-random procedure.

Tests were performed using a simulator on the “affected” test arm; training sessions were performed with the simulator on the contralateral “unaffected” training arm. All groups started with a pretest (day 1) to establish the skills for the participants’ test arms. The Short Interval Training Group then trained for five consecutive days; the Long Interval Training Group trained twice a week. Subsequently, the participants performed a posttest and two retention tests. Both the Short and Long Interval Control Groups, although they underwent a sham training program without the simulator, executed test and training sessions on the same days as the corresponding Training Group.

### Outcomes

The primary outcome measurements were as follows.

1. The movement time, the time in milliseconds from the beginning of the movement until completion of the task. Movement time reflects intermanual transfer effects when the Short and Long Interval Training Groups become faster than the Short and Long Interval Control Groups.
2. The duration of the maximum hand opening in milliseconds while picking up an object. In the course of learning to use a prosthesis, the grasping profile will change. In the beginning, hand opening and hand closing are decoupled, reflecting a stepwise control. After learning, hand opening and closing will become coupled, which shortens the duration of the maximum hand opening [30]. This latter profile looks more like natural grasping.
3. The grip-force control, the difference between the required and the applied grip force in N. Grip-force control has been shown to be a difficult part of learning to handle a prosthesis [30–32] and therefore has high clinical relevance.

### Materials

The myoelectric prosthesis simulator (OIM Orthopedie, Haren, the Netherlands) [33,34] consists of a MyoHand VariPlus Speed (Otto Bock, Duderstadt, Germany), with proportional speed control (15–300 mm/s) and proportional grip-force control (0-±100 N), attached to an open cast, in which the hand is placed. The cast extends into a splint along the forearm that can be secured with a Velcro sleeve. With this, two electrodes are placed on the muscle bellies of the forearm. The prosthesis hand is controlled by changes in electrical activity related to muscle contraction.

The MyoBoy (757M11 Myoboy; 13E200 MyoBock Electrodes; Otto Bock, Duderstadt, Germany) was used to determine the sensitivity of the electrodes.

E-Prime (Psychology Software Distribution, York, UK), an application suite, was used to register movement time.

**Fig 3:**
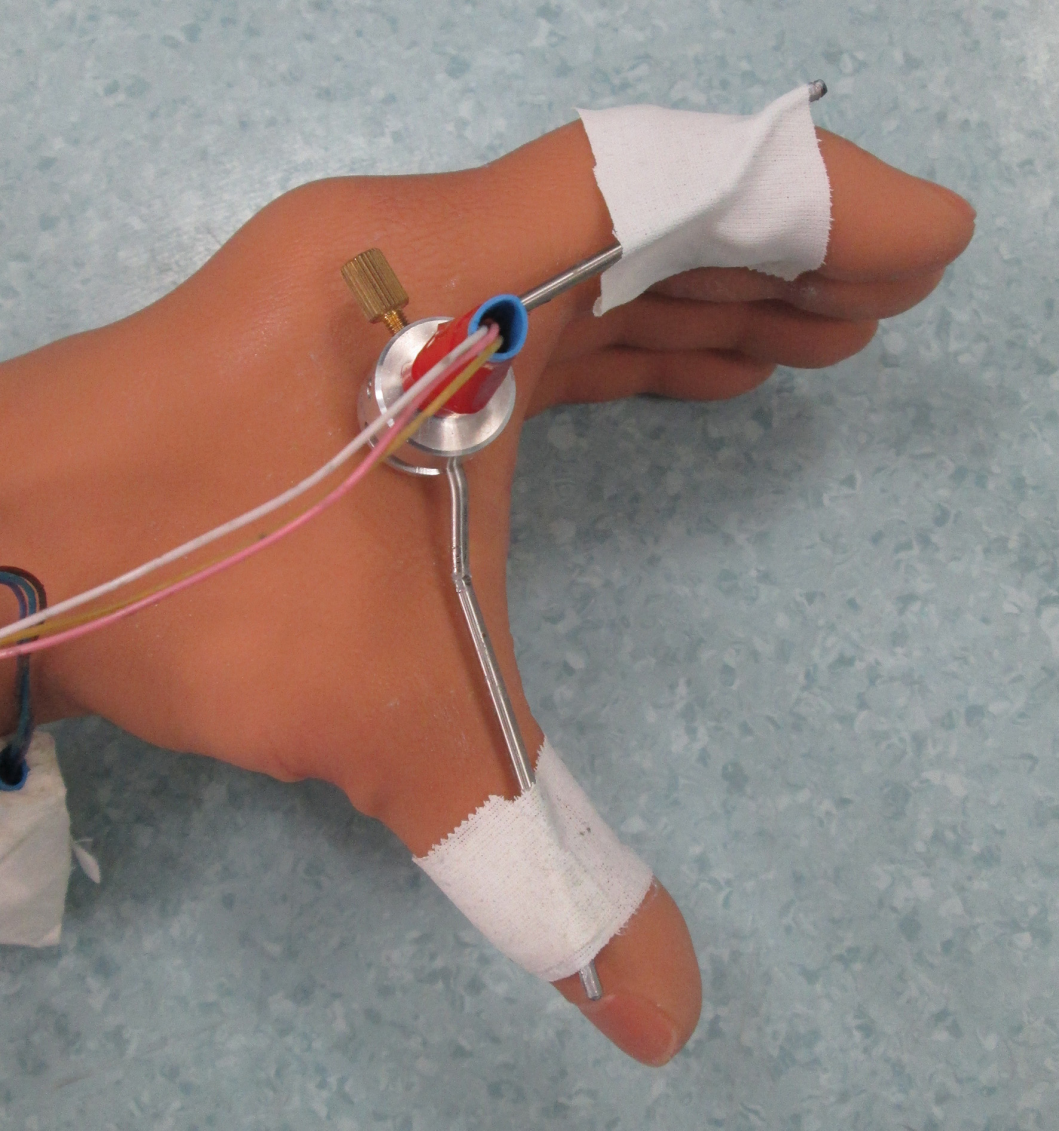
Potentiometer attached to the prosthesis hand.

The hand opening was measured using a potentiometer (Fig 3), with one small rod attached to its base and another rod attached to the wiper (e.g., the moving part of the potentiometer). One of the rods was attached to the thumb and the other to the index finger of the prosthetic hand. The output of the potentiometer was digitally sampled with a 32-channel Porti system (TMSI, Enschede, the Netherlands).

**Fig 4:**
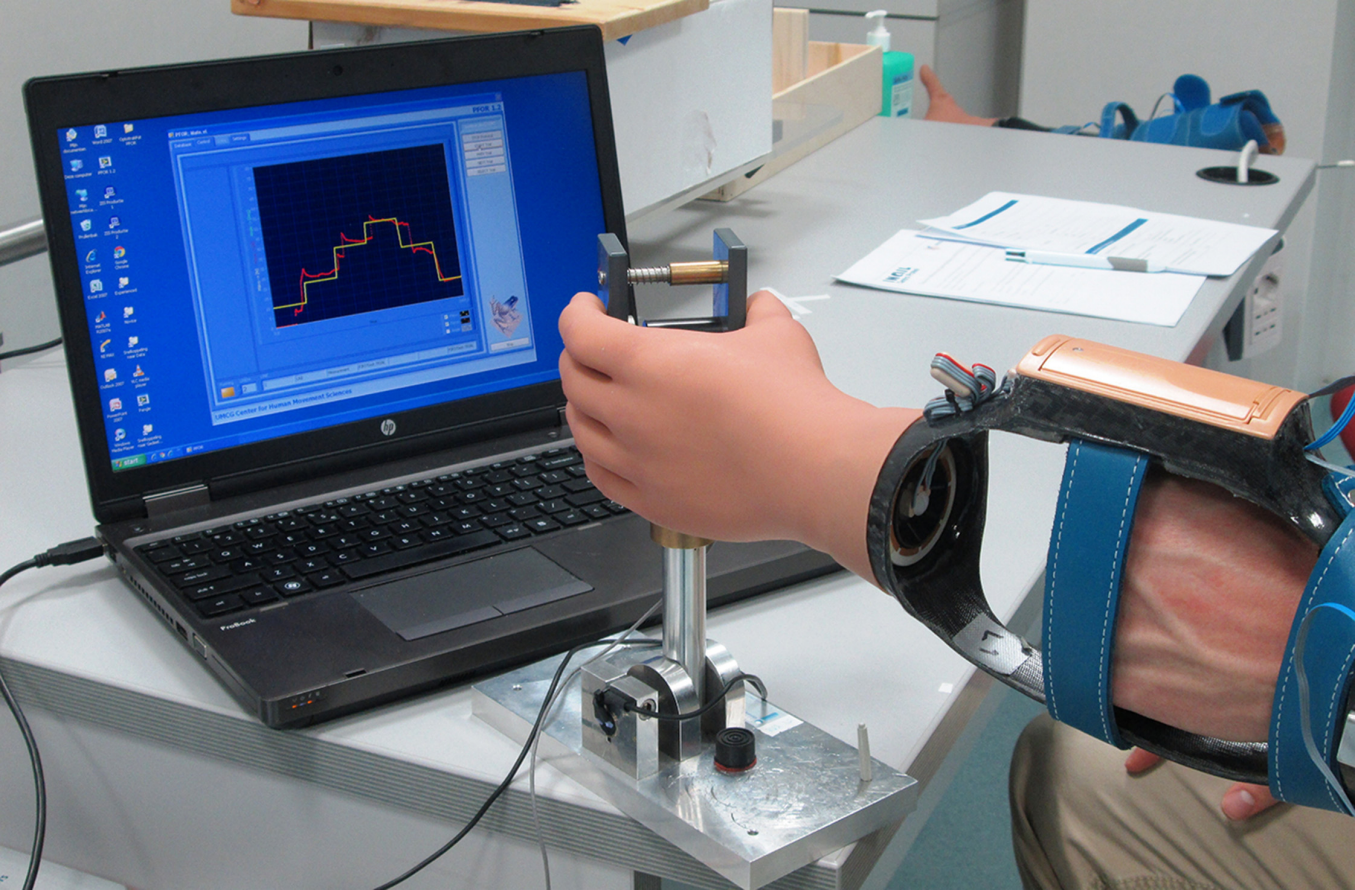
A custom-made program to measure grip force.

A custom-made program created with Labview (display and sample frequency 100 Hz) was used to measure the amount of grip force when pinching a handle (Fig 4) [35]. The handle consisted of two plates with a force transducer (LLB350 Loadcell [Futek]) placed in between [35].

### Interventions

All sessions took place at the university laboratory. The experimenter executing the tests was blinded for the randomization of the participants. The test and training sessions started with a standard procedure to fit the simulator. After palpation of extensor carpi ulnaris and flexor carpi radialis muscle bellies, the electrodes were positioned. Using the MyoBoy, the amplified signal of the electrodes had to exceed a threshold of 1.5 V (high signal), sustained for two seconds. The maximum speed of the hand was set to the default setting of six (double channel control; fast open and slower closing). After the simulator was fitted, the participant was positioned in front of the table with the elbow flexed 90 degrees. Verbal instruction concerning the execution of the tasks was provided.

#### Pretest, posttest, and retention tests

The pretest, posttest, and both retention tests all consisted of a functional and a grip-force control test, executed in random order. The three functional test tasks and the grip-force control test task were all repeated three times. The functional test tasks were based on the three different ways prostheses are used in daily life [36] and were the same as used in our previous study [6]: the mug task (direct grasping), jar-lid task (indirect grasping), and pen-case task (fixating). A computer screen showed which task had to be executed. Before and after each task, the participant pressed the spacebar on a keyboard that was positioned at the test side to measure movement time. During the mug task, the duration of the hand opening was recorded (Table 1).

**Table 1:** Summary of the dependent variables for all test tasks.

Test	Task	Dependent variable
Functional test	Mug task	Movement time (ms)
		Hand opening (ms)
	Jar-lid task	Movement time (ms)
	Pen-case task	Movement time (ms)
Grip-force control test	Tracking task	Deviation of grip-force control (N)

The grip-force control test consisted of a tracking task on a computer screen. Using a custom-made program, the participant had to track a target line shown on the screen. By pressing a handle, the cursor height could be adapted, which was visible as a second line on the screen [35].

We originally planned to also test reaching movements; we decided, however, not to perform this test. This decision was based on a finding in an earlier study, the results of which are as yet unpublished, where no improvement was seen in the reaching movements, not even after a training program specifically intended to improve this. Furthermore, because we used the potentiometer instead of a more advanced movement registration system, we were no longer able to calculate the reaching movement.

#### Training sessions

All groups completed a training program of five days of 30-minute sessions. The Short and Long Interval Training Groups sessions were divided into 20 minutes of functional training and 10 minutes of grip-force control training. During the functional training, participants practiced tasks from the Southampton Hand Assessment Procedure (SHAP) [37]. SHAP consists of twelve abstract object tasks and 14 activities-of-daily-life tasks, and evaluates functionality of hand prostheses. During the grip-force control training, participants practiced different tracking-task patterns.

The Short and Long Interval Control Groups executed a sham training program, which also focused on contractions of wrist muscles but without using the prosthesis simulator. The training consisted of 20 minutes of playing tennis on the Wii, with the participant seated in front of the screen. For the remaining 10 minutes, participants played darts while standing two meters away from the board. This training program was chosen in such a way that the Short and Long Interval Control Groups, like the Short and Long Interval Training Groups, trained the coordination between wrist muscles. Moreover, the selected tasks motivated participants to improve their skill.

### Statistical Analyses

Analyses were executed using Social Package Statistical Science (SPSS) 22.0 software package (SPSS, IBM Corp in Armonk, NY). All outliers that deviated more than three times the standard deviation per test were removed. In analyzing the functional tests, z-scores were used that were calculated for the three test tasks. For all variables, the means for the three trials in each test were calculated. Missing values were replaced by estimated means.

We performed ANCOVAs, where the pretest was used as covariate, to correct for possible differences between groups. Because we were also interested in the effect of the covariates on the between-subjects variables (the training and inter-training interval), we included these tests in the standard full factorial model.

To analyze the *movement times* of the functional tasks a mixed-design ANCOVA was performed with test (posttest, retention test 1, and retention test 2) and task (mug, jar-lid, and pen-case) as within-subject factors, and training (Training Groups and Control Groups) and interval (Short Interval Groups and Long Interval Groups) as between-subject factors. The movement times for each of the tasks at pretest (mug, jar-lid, and pen-case) were used as covariates.

The *duration of the maximum hand opening* was calculated using Matlab R2007a. The angle of the hand opening was first filtered with a 20 Hz cut-off frequency using a recursive second-order Butterworth filter. To obtain the velocity of the hand aperture, the opening profile was differentiated using a three-point difference algorithm. The peak values of this velocity profile were used to determine the hand opening and closing. By searching backward and forward for the first value that was below a threshold of 2.5 cm/s and that stayed there for 50 ms, the end of the first hand opening and start of the hand closing were defined. The period between these points was taken as the duration of the maximum hand opening.

A mixed-design ANCOVA on the means of the duration of the maximum hand opening was performed to examine whether the groups differed, with test (posttest, retention test 1, and retention test 2) as a within-subject factor, training (Training Groups and Control Groups) and interval (Short Interval Groups and Long Interval Groups) as between-subject factors, and the pretest as covariate.

A mixed-design ANCOVA was performed on the *error of the grip-force control* with test (posttest, retention test 1, and retention test 2) as a within-subject factor, training (Training Groups and Control Groups) and interval (Short Interval Groups and Long Interval Groups) as between-subject factors, and the pretest as covariate.

When sphericity was violated, the degrees of freedom were adjusted with the Greenhouse-Geisser correction. A significance criterion of 0.05 was used during the analysis. Post-hoc tests on main effects tested all pair-wise comparisons. All post-hoc tests used a Bonferroni correction and the corrected values are described. Only significant findings are reported.

Two additional analyses were performed. One on the test hand for the variables of movement time, hand opening, and grip-force control. And one where we compared the analysis on the data with the missing values with the analyses with replaced values.

## Results

The participants (25 men, 39 women; mean age 22.75 [±SD = 4.50] years) had a mean laterality quotient of 89 (range 40–100). The lowest score of 40 indicated that all participants were right-handed. Concerning the data regarding the movement times, 3.6% of these data was missing due to outliers (2.0%) and technical problems. In total, 16.5% of the data regarding the duration of maximum hand opening was missing, of which 1.6% was due to outliers and the remaining due to technical problems in the data collection. Concerning the grip-force control data, 2.5% of them were misses, of which 1.2% due to outliers. Analyses with and without missing variables produced similar results.

### Movement time

The mixed-design ANCOVA on movement time (Table 2) showed a main effect for all covariates (the three tasks at pretest) (all P’s<.037). Three interaction effects were significant after correcting for sphericity. An interaction of task and the covariate jar-lid task at pretest (F_1.79,91.31_ = 4.15, p = .022), an interaction of task, inter-training interval, and the covariate pen-case task at pretest (F_1.79,91.31_ = 4.31, p = .020), and an interaction of session, task, and the covariate jar-lid task at pretest were also significant (F_3.35,170.80_ = 3.01, p = .027). These interactions reveal effects for two of the covariates (the jar-lid and pen-case task at pretest) on the data. Because differences between tasks were expected but were not of primary concern for the present study, these effects were not discussed any further.

**Table 2:** Means (Confidence Interval) for movement times and hand opening (in milliseconds) and deviation in grip-force control (in Newtons) for the groups per test.

		Short Interval		Long Interval	
Variable	Test	Training	Control	Training	Control
**Movement time (ms)**	**Pretest**	6719 (5807–7630)	6718 (5807–7630)	7369 (6458–8281)	7914 (7003–8825)
	**Posttest**	4633 (3997–5269)	5304 (4668–5940)	4693 (4057–5329)	5834 (5198–6470)
	**Retention test 1**	4145 (3641–4649)	4572 (4068–5076)	4104 (3597–4605)	5167 (4662–5671)
	**Retention test 2**	3969 (3509–4428)	4176 (3716–4635)	4064 (3604–4523)	4932 (4473–5392)
**Hand opening (ms)**	**Pretest**	903 (739–1067)	791 (627–955)	909 (745–1073)	900 (736–1064)
	**Posttest**	739 (592–885)	655 (508–801)	707 (561–853)	731 (585–877)
	**Retention test 1**	552 (386–718)	664 (498–830)	607 (441–773)	695 (529–861)
	**Retention test 2**	661 (505–816)	440 (285–596)	595 (440–751)	626 (470–781)
**Grip-force control (N)**	**Pretest**	9.45 (7.06–11.84)	10.88 (8.49–13.27)	10.54 (8.14–12.94)	9.73 (7.34–12.12)
	**Posttest**	6.24 (4.89–7.59)	8.22 (6.87–9.56)	5.98 (4.64–7.33)	7.40 (6.05–8.74)
	**Retention test 1**	5.73 (4.58–6.88)	6.93 (5.78–8.08)	6.08 (4.93–7.23)	6.62 (5.47–7.77)
	**Retention test 2**	5.58 (4.59–6.56)	6.97 (5.99–7.96)	6.45 (5.46–7.43)	6.31 (5.32–7.30)

**Fig 5:**
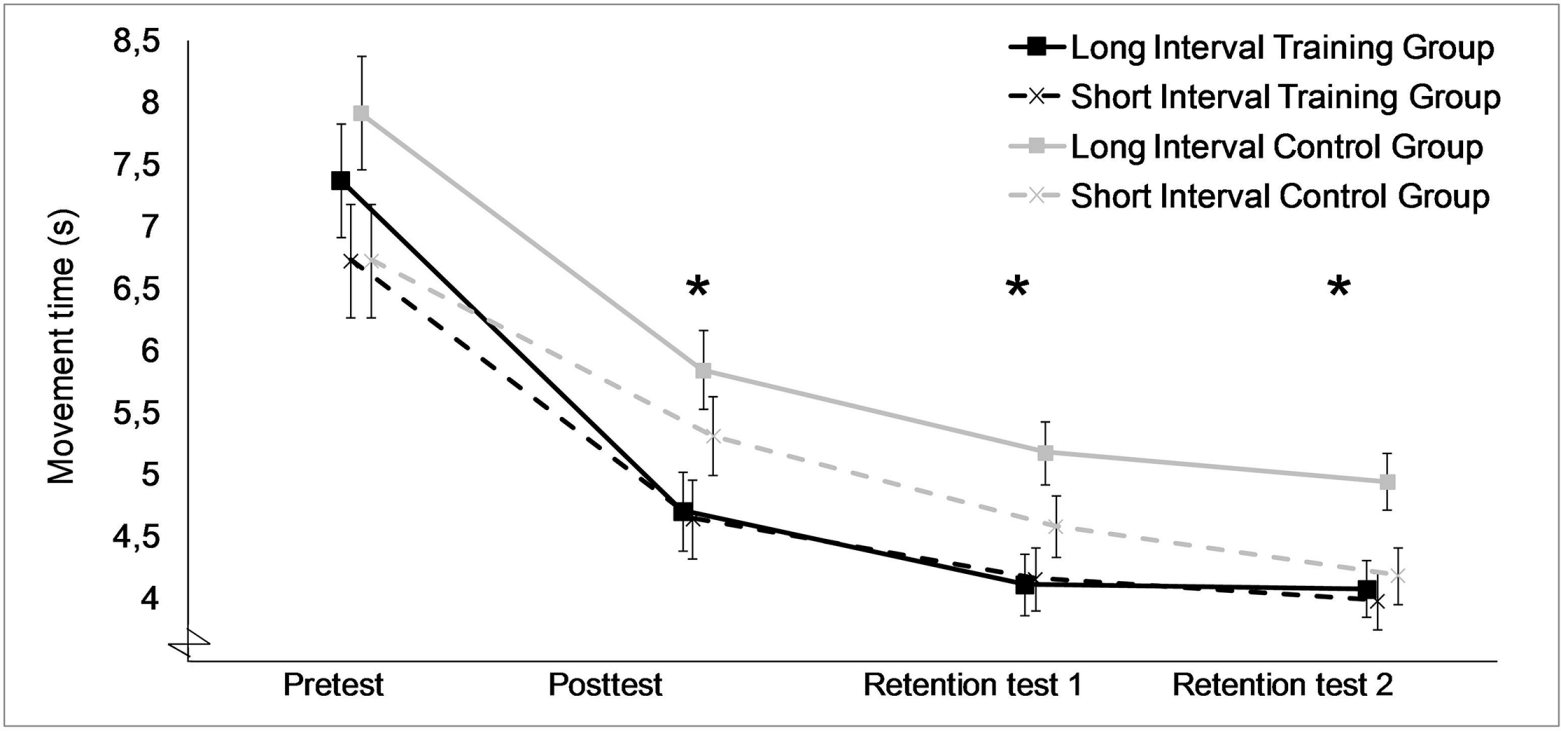
Mean movement times (±SE) of the functional tests in seconds. Note that the figure shows real movement times, while the analyses were performed on the z-scores. * shows the significant difference (P<.001) between the means of both Training Groups and both Control Groups.

Improvement over tests, corrected for sphericity violations, was found to be significant (F_1.72,87.81_ = 6.04, P = .005). Post-hoc tests on the main effect of session, using a Bonferroni correction (α = 0.05/3 = 0.017), showed a significant improvement between the posttest and retention test 1 (P<.001), and between the posttest and retention test 2 (P<.001), revealing an improvement in performance after the training program has ceased. The ANCOVA on movement time further showed an effect of training programs (F_1,51_ = 6.79, P = .012), where after training the Training Groups were faster than the Control Groups (Fig 5). This means that the Training Groups improved more after training and the effect of the intermanual transfer training is present.

### Duration of the maximum hand opening

A main effect of the covariate (pretest) was found, revealing an influence of the pretest on the findings (F_1,57_ = 27.79, P<.001). The longer the duration of the maximum hand opening on the pretest, the longer was the duration on the posttest and retention tests. The ANCOVA on the duration of the maximum hand opening did not show significant intermanual transfer or inter-training interval effects.

### Deviation in force control

The larger the deviation in the force control on the covariate (pretest) the larger was the deviation on the other tests (F_1,57_ = 4.94, P = .030). Nevertheless, we did not find training or inter-training interval effects.

### Additional analyses

The analysis on the test hand for the variables of movement time, hand opening, and grip-force control did not show significant differences for the preferred or non-preferred test hand. This was what we expected from previous studies measuring the effect of the test hand in prosthetic training [5–7].

The comparison on the data with the missing values with the analyses with replaced values only showed a difference for the main effect of task on movement time, (corrected for sphericity violations F_1.75,80.39_ = 3.34, p = .047). This effect could be neglected because it was not of importance for the research question; furthermore, the post-hoc tests using a Bonferroni correction (α = 0.05/3 = 0.017) and comparing all tasks with each other did not reveal any difference (All P’s>.052).

## Discussion

Intermanual transfer effects were demonstrated in able-bodied persons using a prosthesis simulator. After training the “unaffected” hand, the movement times of the “affected” hand improved, which is consistent with the literature [5–7]. This intermanual transfer effect was not influenced by the length of the inter-training interval. Intermanual transfer was not demonstrated in the duration of the maximum hand opening or in the grip-force control. Effects of the covariate (pretest) showed influence on all dependent variables. The implications of these findings in terms of rehabilitation practice are that persons with an upper-limb amputation, who train prosthetic skills using intermanual transfer, can train at their own preferred pace; no additional benefit is to be expected from training on a daily basis.

Inter-training intervals which were lengthened to 24 hours positively influenced the learning of several tasks [16–18], including one that used intermanual transfer [21]. To better imitate rehabilitation practice, we applied inter-training intervals of at least 24 hours. The literature regarding these longer intervals is less consistent. It seems that with longer inter-training intervals the learning effect stabilizes and, at a certain point, even decreases [18,22,24]. This stabilization indicates that the optimal learning effect remains consistent over a range of inter-training interval durations. Thus, if our two inter-training intervals were within this stabilization period, differences could not be found. For persons with an upper-limb amputation, these findings suggest that training sessions on consecutive days or twice a week might give comparable results. Based on the literature, it might be that transfer effects decrease if inter-training intervals that are shorter (<24 hours) or longer than the ones in this study are used.

It should be mentioned that the Short and Long Interval Control Groups also improved over time in terms of prosthesis skills on the “affected” side, even though they did not train with the prosthesis simulator. These improvements are probably caused by having performed the test tasks repeatedly with the prosthetic simulator on the test side. From our findings, it is clear that the intermanual transfer effect does contribute to some degree to any improvement. In our earlier studies we found the intermanual transfer effect to be small [6,7]. The small effect is to be expected because the actual side that needs to improve is not trained directly, and, of course, direct training of an arm results in the largest effect. Therefore, the effects of intermanual transfer are expected to be small. Nevertheless, we assume the size of the effect to be clinically important, because, as a result of the training, a patient will start using the prosthesis at a higher level, and this may encourage motivation.

The first retention test of the Long Interval Groups and the second retention test of the Short Interval Groups took place on the same day. However, when comparing both tests on day 17, it might be expected that the Short Interval Groups will perform better, because they have performed the tests twice, where the Long Interval Groups performed the tests once. Therefore, we compared results of both first retention tests of the Long Interval Group and the Short Interval Group with each other.

What needs to be mentioned when focusing on the retention tests is that the optimal spacing of training intervals is also related to the retention interval. A meta-analysis study showed that in recall tasks with longer retention intervals, the inter-training interval leading to better training results appeared to be longer [18]. In the current study, the length of the retention interval was about the same as the inter-training interval. We therefore suppose that the length of the retention interval influenced both inter-training intervals (short and long) in a comparable way. For persons with an amputation, who are training prosthesis skills using intermanual transfer, the retention test resembles the moment when the person receives his/her prosthesis. It may be possible to lengthen the inter-training interval (while avoiding reaching the point at which the effects decrease), depending on how long it takes before the patient obtains the prosthesis.

The intermanual transfer effect found on the movement times was not replicated in the duration of the maximum hand opening. We expected the duration to diminish over trials, as the performance of the participant improved [31]. Our results might be explained by the occurrence of bumpy trials [38], where the hand is opened partly and then, before closing, opened further. More training sessions might be necessary to overcome the sub-opening of the hand and to find possible intermanual transfer effects. Thus, although the hand opening did not transfer, the improvement in movement time does reflect improvement in skill, and this might be relevant for how participants experience performing the prosthetic task.

The intermanual transfer effect and the influence of the inter-training interval on grip-force control were not present. In our previous studies on prosthetic handling, we did not find intermanual transfer effects on grip-force control tasks either [6,7]. Contrary to the previous studies, the current study contained specific grip-force control training. It is known that grip-force control with a prosthesis is hard to learn and takes more than five sessions, which may explain this finding [30–32]. It might be that more training sessions or longer training sessions are necessary to measure improvement.

One of the strengths of this study is the use of Control Groups executing a sham training program, instead of the crossover design applied in most studies on intermanual transfer [11,14,39–45]. The sham training program consisted of a functional part executed with the same wrist muscles as used during prosthetic simulator training. This made it possible to exclude the factor that training the coordination of the involved muscles in itself might be responsible for intermanual transfer effects. The results imply that training with the prosthetic simulator leads to the revealed intermanual transfer effect.

A limitation of our study is that the experiments were performed using able-bodied persons; measuring over 60 patients obtaining a myo-electric prosthesis for the first time was unfeasible. However, using able-bodied participants instead of amputee patients may have affected the results. After an upper-limb amputation, neural plasticity causes changes in the brain [46]. These changes not only take place in the cortex but occur also in other parts of the brain, like the corpus callosum [47]. The corpus callosum, which connects the two brain hemispheres, is considered to play a major role in intermanual transfer effects [48]. As such, the extent to which intermanual transfer is possible may differ in able-bodied individuals as compared to persons with an amputation. Unfortunately, as far as is known by the researchers, no literature on this topic is available. Nevertheless, we expect the learning effects in patients to be, to a large degree, comparable to those in able-bodied participants, because learning to handle a myo-electric prosthesis simulator has been found to be similar to learning to handle an actual myo-electric prosthesis [31].

## Conclusions

Intermanual transfer effects were present only in the movement time after prosthesis training; we did not find differences in inter-training intervals. Persons with an upper-limb amputation are advised to use intermanual transfer techniques during the period they are waiting for the prosthesis to be manufactured. They can train with a frequency ranging from every day to twice a week.

## Supporting Information

S1 Checklist CONSORT Checklist. (DOC)

S1 Protocol Adapted Study Trial Protocol. (PDF)
