# Supplementary material for: Influence of Inter-Training Intervals on Intermanual Transfer Effects in Upper-Limb Prosthesis Training: A Randomized Pre-Posttest Study
Source: PLoS One. 2015 Jun 15;10(6):e0128747. doi: 10.1371/journal.pone.0128747 (PMC4468151; doi:10.1371/journal.pone.0128747)
Supplement: S1 Protocol — (PDF) [file pone.0128747.s002.pdf]

RESEARCH PROTOCOL

Optimizing the intermanual transfer effects after training with a prosthetic simulator

TABLE OF CONTENTS

1. INTRODUCTION AND RATIONALE 5
2. OBJECTIVES 6
3. STUDY DESIGN 6
4. STUDY POPULATION 7
   4.1 Population (base) 7
   4.2 Inclusion criteria 7
   4.3 Exclusion criteria 7
   4.4 Sample size calculation 8
5. TREATMENT OF SUBJECTS 8
   5.1 Investigational product/treatment 8
   5.2 Use of co-intervention (if applicable) 8
   5.3 Escape medication (if applicable) 8
6. INVESTIGATIONAL MEDICINAL PRODUCT 8
7. NON-INVESTIGATIONAL PRODUCT 8
8. METHODS 8
   8.1 Study parameters/endpoints 8
      8.1.1 Main study parameter/endpoint 8
      8.1.2 Secondary study parameters/endpoints (if applicable) 9
      8.1.3 Other study parameters (if applicable) 9
   8.2 Randomisation, blinding and treatment allocation 9
   8.3 Study procedures 9
   8.4 Withdrawal of individual subjects 12
      8.4.1 Specific criteria for withdrawal (if applicable) 12
   8.5 Replacement of individual subjects after withdrawal 12
   8.6 Follow-up of subjects withdrawn from treatment 12
   8.7 Premature termination of the study 13
9. SAFETY REPORTING 13
   9.1 Section 10 WMO event 13
   9.2 AEs, SAEs and SUSARs 13
      9.2.1 Adverse events (AEs) 13
      9.2.2 Serious adverse events (SAEs) 13
   9.3 Annual safety report 13
   9.4 Follow-up of adverse events 13
   9.5 Data Safety Monitoring Board (DSMB)/Safety Committee 14
10. STATISTICAL ANALYSIS 14
   10.1 Primary study parameter(s) 14
   10.2 Secondary study parameter(s) 14
   10.3 Other study parameters 14
   10.4 Analysis (if applicable) 14
11. ETHICAL CONSIDERATIONS 14
   11.1 Regulation statement 14
   11.2 Recruitment and consent 14

11.3 Objection by minors or incapacitated subjects (if applicable)................................14
11.4 Benefits and risks assessment, group relatedness...15
11.5 Compensation for injury 15
11.6 Incentives (if applicable)...15
12. ADMINISTRATIVE ASPECTS, MONITORING AND PUBLICATION........................15
12.1 Handling and storage of data and documents 15
12.2 Monitoring and Quality Assurance..15
12.3 Amendments...15
12.4 Annual progress report...15
12.5 End of study report...15
12.6 Public disclosure and publication policy 16
13. STRUCTURED RISK ANALYSIS..16
13.1 Potential issues of concern 16
13.2 Synthesis 16

LIST OF ABBREVIATIONS AND RELEVANT DEFINITIONS

**ABR**	**ABR form (General Assessment and Registration form) is the application form that is required for submission to the accredited Ethics Committee (ABR = Algemene Beoordeling en Registratie)**
**AE**	**Adverse Event**
**AR**	**Adverse Reaction**
**CA**	**Competent Authority**
**CCMO**	**Central Committee on Research Involving Human Subjects**
**CV**	**Curriculum Vitae**
**DSMB**	**Data Safety Monitoring Board**
**EU**	**European Union**
**EudraCT**	**European drug regulatory affairs Clinical Trials GCP Good Clinical Practice**
**IB**	**Investigator's Brochure**
**IC**	**Informed Consent**
**IMP**	**Investigational Medicinal Product**
**IMPD**	**Investigational Medicinal Product Dossier**
**METC**	**Medical research ethics committee (MREC); in Dutch: medisch ethische toetsing commissie (METC)**
**(S)AE**	**Serious Adverse Event**
**SPC**	**Summary of Product Characteristics (in Dutch: officiële productinfomatie IB1-tekst)**
**Sponsor**	**The sponsor is the party that commissions the organisation or performance of the research, for example a pharmaceutical company, academic hospital, scientific organisation or investigator. A party that provides funding for a study but does not commission it is not regarded as the sponsor, but referred to as a subsidising party.**
**SUSAR**	**Suspected Unexpected Serious Adverse Reaction**
**Wbp**	**Personal Data Protection Act (in Dutch: Wet Bescherming Persoonsgevens)**
**WMO**	**Medical Research Involving Human Subjects Act (Wet Medisch-wetenschappelijk Onderzoek met Mensen**

SUMMARY

Rationale: To improve the rate of use of prosthetic devices in adults with an upper limb amputation intermanual transfer might be helpful. Intermanual transfer is the ability to transfer motor skills from one, trained side to the other side (Hicks, 1983). This can be used in upper limb amputees by training the unaffected arm while waiting for the prosthesis to be fitted. Especially because it is assumed that training starting early after the amputation will lead to better acceptance and improved prosthetic handling (Malone, Fleming et al. 1984). Due to intermanual transfer, the prosthetic skills of the affected arm will then improve. Intermanual transfer effects were demonstrated to be present in myo-electric (Romkema, Bongers et al. 2013) and body-powered prosthesis use (Weeks, Wallace et al. 2003). However, it is unclear how the training program should be like to obtain the largest effects. The question rises how the training should be spaced over time for the best results.

Objective: To compare different training intensities to be able to measure which training has the largest effects.

Study design: non-blinded randomized trial.

Study population: 64 non-amputated adults.

Intervention (if applicable): Four groups of 16 participants train to use a prosthetic simulator for 20 min during 5 days and are tested on their skills with a prosthetic simulator. The prosthetic simulator mimics the functioning of a real prosthesis but can be worn by able-bodied participants and at the sound side of an amputee patient. The prosthesis simulator places a prosthetic hand in front of the sound hand.

Main study parameters/endpoints:

- Grip force control: mean deviation of the asked force in N.
- Reaching: mean deviation of the straight path towards the aim in mm
- Grasp: shape of the grasp profile; plateau duration in s.
- Movement time: time taken to execute the movement in s.

Nature and extent of the burden and risks associated with participation, benefit and group relatedness:

All participants will use the prosthetic simulator. This simulator mimics a real prosthetic device and can be worn over a sound arm. Because of the use of this simulator we are able to test more participants than only the few recently amputated patients. Importantly, all the measurements are non-invasive and the use of a prosthetic simulator is not different from wearing a regular prosthesis. Therefore, the risks associated with participation can be considered negligible and the burden can be considered minimal.

1. INTRODUCTION AND RATIONALE

To improve the rate of use of prosthetic devices in adults with an upper limb amputation we have been using intermanual transfer. Intermanual transfer implies that when you learn a motor task with one arm, not only that arm improves, but also the arm at the other side becomes better in the specific task (Hicks, Gualtieri et al. 1983, Karni, Meyer et al. 1998, Kumar, Mandal 2005, Lee, Hinder et al. 2010, Mier, Petersen 2006, Pereira, Raja et al. 2011). The untrained side thus benefits from the trained side. In other words, the effect of intermanual transfer is that the prosthetic skills of the affected arm will improve.
The intermanual transfer effect is shown to be present in body-powered (Weeks et al., 2003) and also in myo-electric prostheses (Romkema, Bongers et al. 2013). With able-bodied participants we showed that after training the ‘unaffected’ side using a prosthesis simulator, the level of skills at the ‘affected’ side increased. This effect can be useful in rehabilitation after an upper limb amputation, because the training can be started earlier. It is assumed that training immediately after the amputation will lead to better acceptance and prosthetic handling (Malone, Fleming et al. 1984). It is found that training should start within one month after the amputation to achieve maximum success (Atkins 1992, Dakpa, Heger 1997, Gaine, Smart et al. 1997). Though in this period often the wounds are not healed yet and the prosthesis is not finished. Using a prosthetic simulator the training at the sound side can start early what might lead to better acceptance and higher prosthesis skills due to effects of intermanual transfer. This might decrease rejection rates of prosthesis devices rejected.

To be able to train the unaffected arm we make use of a prosthetic simulator. With this simulator it is possible to mimic a myo-electric prosthesis. A prosthetic hand can be opened and closed with a motor driven by electrical signals that are produced by muscle activation. The simulator is placed over the arm, and the prosthetic hand is placed before the sound hand (Figure 1) and then operates in the same way as the prosthesis. The training with the simulator is therefore comparable to the training with the myo-electric prosthesis.

In our earlier study (Romkema, Bongers et al. 2013) where we demonstrated the effect of intermanual training in prosthetic learning we used functional tasks. We found that the movement times increased after a five-day training program.

We now would like to optimize the spacing of the training program. It is generally known that variations in spacing affect the effectiveness on training (Kornmeier, Sosic-Vasic 2012, Donovan, Radosevich 1999, Schmidt, Lee 2005, Shea, Lai et al. 2000) For instance, Donovan (1999) found in a meta-analyses of 63 studies, that training in spaced practice conditions leads to significantly higher performance than training in massed practice conditions. Literature on the spacing of training sessions is mostly focused on intertraining periods within 24 hours. Because in this literature the longer intervals (i.e., up to 24 hours) are found to have the largest effects (Siengsukon, Boyd 2009, Kornmeier, Sosic-Vasic 2012, Shea, Lai et al. 2000, Goedert, Miller 2008, Hussain, Sekuler et al. 2009) and because such intertraining time intervals are realistic for rehabilitation we will use intervals of at least 24 hours. It is currently unknown what the effect is of longer time intervals. The current study will examine these longer time intervals because in rehabilitation practice they are often more practical. In an earlier pilot of our group, measuring three people training with the simulator in different intervals, we found the largest effect on the every-day training. This suggests that we might get the largest effect with an every-day training.

In conclusion, with this study we aim to optimize the intermanual transfer effects in prosthetic use that we have shown to be present in earlier research. For this we compare different time intervals between training sessions (i.e., spacing of the training). This research will be done with able-bodied participants. Including able-bodied participants will mean that we do not have to bother patients who have just been amputated.

2. OBJECTIVES

The objective of our study is to compare different training intensities to be able to measure what training has the largest effects.

3. STUDY DESIGN

The experiment aims to reveal the effect of spacing on the intermanual transfer. As found in the available literature, for different tasks, the largest effects are found with a period of minimally 24 hours, the longest period that is tested, between training sessions. Though, the topic of spacing has not received a lot of attention in training motor skills and for periods longer than 24 hours. The sparse evidence revealed that the optimal training effects depend on the nature of the task and on the combination of time interval between the training and the time till the retention test. For this study we therefore choose to use two different time intervals with a minimum of 24 hours. The first group trains daily, the second group trains with two and three days intervals between the training sessions. The training tasks will be chosen based on the previous experiment; the tasks with the largest effects will be used. Two control groups are added both following one of the training shemes of the training groups. These groups will execute a sham training where the wrist muscles are used though the prosthesis is not worn. The tests consists of a pretest, posttest and retention test (seven days after ending the training), to be able to measure whether there were learning effects and whether these effects remained (see 7.3 for an extensive explanation). All tests consist of the same tasks; functional, grip force control, reaching and grasping tasks. Half of the

participants will train their dominant hand and half will train their non-dominant hand. Apart from the three tests, as used in the last experiment, there will be an extra retention test. The first retention test takes place on day 17, so that the first retention test is at the same time interval after the first training for all the spacing regimes. The second retention test is conducted two weeks after the last training session. This will make it possible to show the effects of the different intervals till two weeks after the training.

Table 2. Study design of the second experiment on spacing

Healthy adults								
Spacing	**Short interval**		**Short control**		**Long interval**		**Long Control**	
Participants	*8 men, 8 women (½ dom, ½ n-dom)*		*8men, 8 women (½ dom, ½ n-dom)*		*8 men, 8 women (½ dom, ½ n-dom)*		*8 men, 6 women[illegible] dom, ½ n-dom)*	
**Day**	**Test**	**Practice**	**Test**	**Practice (sham)**	**Test**	**Practice**	**Test**	**Prac[illegible] (sha[illegible]**
1 (Mon)	Pretest	30 min	Pretest	30 min	Pretest	30 min	Pretest	30 m[illegible]
2 (Tue)		30 min		30 min				
3 (Wed)		30 min		30 min				
4 (Thurs)		30 min		30 min		30 min		30 m[illegible]
5 (Fri)	Posttest	30 min	Posttest	30 min				
8 (Mon)						30 min		30 m[illegible]
9 (Tue)								
10 Wed)								
11 (Thurs)						30 min		30 m[illegible]
12 (Fri)								
15 (Mon					Posttest	30 min	Posttest	30 m[illegible]
16 (Tue)								
17 (Wed)	Retention test		Retention test		Retention test		Retention test	
19 (Fri)	Retention test		Retention test					
24(Wed)								
29 (Mon)					Retention test		Retention test	

.

4. STUDY POPULATION

4.1 Population (base)

64 non-amputated adults

4.2 Inclusion criteria

(1) Normal or corrected to normal sight
(2) Right-handed
(3) Aged 18 till 40

4.3 Exclusion criteria

(1) Neurological problems concerning upper extremity or torso
(2) Motor problems concerning upper extremity or torso

(3) Earlier experience with a prosthetic simulator
(4) Limited sight despite correction

4.4 Sample size calculation

For the second experiment the sample size calculation is done based on the data a previous experiment. The test tasks of the experiment are also used in the first experiment. The participants of both experiments will train on 5 days. We used the pretest and retention test of the force control task to decide what the expected differences are. Using g power we made an estimation of the amount of participants necessary within each group to reach a power of 0.8. Here for we used single side testing with an alfa of 0.05.
Because of the changes for this experiment we can not give an exact estimation. The training will be longer and the tests shorter. We therefore expect the effects to be larger. Though, because we do not have different training tasks but only a difference in spacing we would expect the differences to be smaller.
With the described method we found that we need to have 17 participants in each group (table 5). We would like to make this 16 participants to get an equal distribution of men/women and dominant/not dominant test hand per group.

Tabel 5

Sample size per group

		power	pretest	retentietest
Task	Effect size	0.8	deviation	Deviation
Force control	.89	17	7.39	5.04

5. TREATMENT OF SUBJECTS

5.1 Investigational product/treatment

The participants learn to use the prosthetic simulator during training sessions. These sessions take place on five days to promote learning. Each training session will take thirty minutes.

5.2 Use of co-intervention (if applicable)

Not applicable

5.3 Escape medication (if applicable)

Not applicable

6. INVESTIGATIONAL MEDICINAL PRODUCT

Not applicable

7. NON-INVESTIGATIONAL PRODUCT

Not applicable

8. METHODS

8.1 Study parameters/endpoints

8.1.1 Main study parameter/endpoint

- Grip force control: mean deviation of the asked force in N is measured in the grip force control tasks.
- Reaching: mean deviation of the straight path towards the target in mm is measured in the reaching task.
- Grasp: length of plateau phase (maximal hand opening) in seconds is measured in the grasping task.
- Movement time: time taken to execute the movement in seconds is measured in the functional task.

8.1.2 Secondary study parameters/endpoints (if applicable)
Not applicable

8.1.3 Other study parameters (if applicable)
Not applicable

8.2 Randomisation, blinding and treatment allocation
The able-bodied adults will be randomly assigned to one of the four groups (experimental and control groups). The number of participants that train with their dominant and non-dominant hand will be equal for both sexes.

8.3 Study procedures
Prior to the start of the experiment all participants sign an informed consent and it will be explained to them that they can stop with the experiment at any time, without giving a reason.

The training program that shows the best results in an earlier study will be used for this experiment. Here only the intensity of the training will be changed over the different groups.

In the experiment the training and test tasks focusing on the same aspects differ from each other. This is to resemble a rehabilitation setting. Not only a single task but the prosthetic skill needs to be learned. In choosing the tasks for the training and test sessions we also take into account the complexity. A relative complex task is used for the training because then the effects on the other arm (of a simpler task) are assumed to be more prominent.

<u>Materials</u>
The myo-electric simulator is developed to closely resemble a myo-electric upper extremity prosthesis for a below-elbow amputation (see Figure 1). The simulator consists of a myo-electric hand, the MyoHand VariPlus Speed® of Otto Bock, attached to an open cast in which the hand can be placed. The cast extends into a splint along the forearm, adjustable in length. The splint can be attached to the arm using a self-adhesive (Velcro) sleeve. The prosthetic hand has proportional speed control (15-399 mm/s) and proportional grip force control (0-±100 N). The hand is controlled by changes in electric muscle activity, detected by 2 electrodes that are placed on the extensors and flexors in the forearm. The exact positions of these electrodes are determined after palpation of the most prominent contraction of muscle bellies of the extensors and flexors. Subsequently, these locations are marked to place the electrodes. The position of the electrodes is then optimized using Otto Bock PAULA®. Hand opening is accomplished by activity of the extensors, while the hand is closed by activity of the flexors. To mimic the use of a prosthesis as closely as possible, the participants are instructed to make minimal movement with the hand, because when one is amputated, the muscles can contract only isometrically.

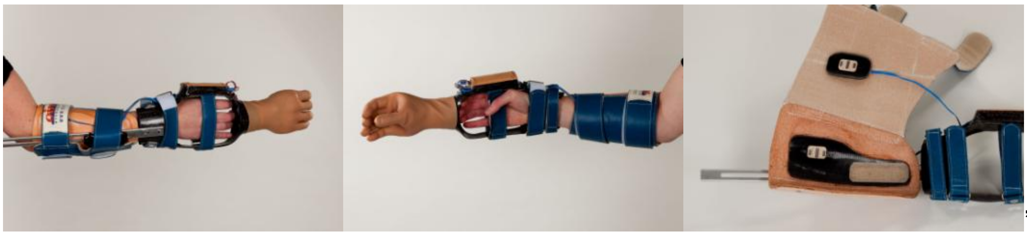
,

Figure 1 a, b and c. The myo-electric simulator, dorsal, volar side and the electrodes inside.

Otto Bock Paula® (Figure 2) is used in conjunction with 757M11 MyoBoy® with USB connection to a PC for the fitting of the electrodes of the simulator. PAULA stands for Prosthetists' Assistant for Upper Limb Architecture, and is used by prosthetists to evaluate myo-signals and further selection and design of the prosthesis.

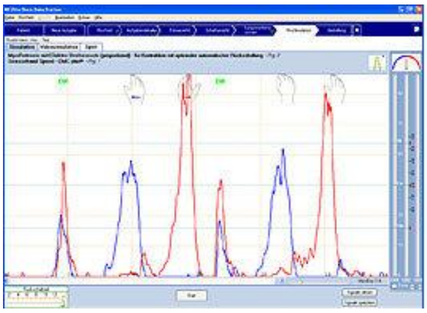

Figure 2. PAULA software on the computer screen, displaying myo-electric signals.

Tests

As described above three aspects of prosthetic performance determine prosthetic skill. The training programs are focusing on one of the three skills or a combination of all of them (functional). The test tasks differ from the training tasks, though they are based on the same skills. The four test tasks (one for each skill) are used to administer the pretests, posttests and one or two retention tests. During the tests the simulator is worn on the test hand (the 'affected' hand) or, in the case of a patient, the prosthesis is used. During training the simulator is worn on the other, 'unaffected' hand. Below, the four test tasks are described in detail.

<u>Grip force control test tasks</u>

Grip force control in this study is the control of the grip force executed on an object that is grasped by the prosthetic hand. Transfer of force control is found to be possible, though it seems to depend on the situation. Maximal force is shown to transfer in different situations, for example (Farthing, Krentz et al. 2011) grip force control and (Lee, Carroll 2007) maximal acceleration of finger abduction. Transfer of proportional isometric force is only shown between limbs on two sides of the body, not between upper and lower limbs (Christou, Rodriguez 2008). Control of force referred towards the environment (Bensmail, Sarfeld et al. 2010, Chang, Flanagan et al. 2008, Teixeira 2000), like in lifting objects, showed better results than force referred towards maximal output, like giving a percentage of the maximal force (Christou, Rodriguez 2008, Park, Shea 2002).

In an earlier study we found that after training only functional tasks in prosthetic training, force control of the untrained hand did not improve (Romkema, Bongers et al. 2013). It is therefore assumed that force control needs to be trained specifically.

We will therefore use a tracking task. In the tracking task a pattern on the screen needs to be followed for 30 seconds by pressing a handle with the prosthetic hand (Figure 4). The pattern consists of different levels of absolute forces (ranged 5 – 45 N) that vary in a blocked pattern. Each amount of force needs to be hold for two seconds. After each trail the participant is allowed to take a break for a few seconds. The course of the pattern appears slightly (200 ms) before the subject has to produce the force. The pattern starts with a line of three seconds of a force of ten Newton, to make sure that participants are able to position the prosthetic hand on the handle, and that all participants have the same starting position. After these first three seconds the blocked pattern starts.

The dependent variable for this test is the mean deviation of the asked force in N.

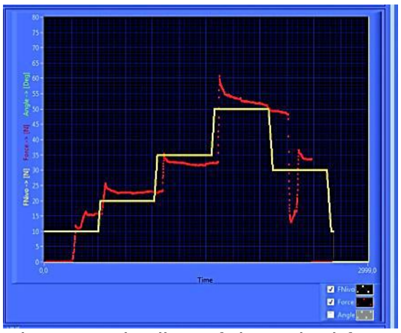

Figure 4. The line of the asked force (yellow) and produced force (red) that can be seen on the computer screen during the tracking task.

<u>Functional test tasks</u>

The functional tasks consist of three object manipulation tasks, as described by Bouwsema (2008). The tasks are based on the three different ways the prosthesis is handled according to van Lunteren et al. (van Lunteren, van Lunteren-Gerritsen et al. 1983); direct grasping, indirect grasping and fixating. In the 'pick-up mug task' the participant has to pick up a mug at the handle with the simulator and to place it 25 cm above the table on a shelf. In the 'lid-off jar task' a jar is picked up by the sound hand at the start and has to be handed over to the simulator, the lid had to be removed by turning it with the sound hand. In the 'zipper task' a pencil case is hold with the simulator at the start position and then the zipper is opened with the sound hand. The dependent variables measured in these tests are the time taken to execute the movement in seconds and the time between the starting signal and the actual start in seconds.

Training

During the training sessions of the experimental groups the simulator is worn on the training hand ('unaffected' hand). All training programs are executed during 20 minutes.

Grip force control training tasks

To train the control of force three tasks are used: the tracking, the matching and the object task. The tracking task is similar to the test task. A pattern on the screen is followed by squeezing a handle. The pattern now varies, blocked and sinus patterns are used.

For the matching task a handle is squeezed as fast as possible until the amount of force shown on the screen is reached. The force needs to be hold for ten seconds. The amount of force will be shown with a cursor on a line and differs in a range between 5-45 N. After each ten trails the participant will be allowed to take a short break.

In the object tasks deformable objects (Romkema, Bongers et al. 2013) are used. The deformable objects have springs with 5 different resistances. Participants have to pick up objects while trying to compress them as minimally as possible.

Functional training tasks

It contains of tasks from the Southhampton hand assessment procedure ((Light, Chappell et al. 2002):

Sham group training tasks

The control group executes sham training.

<u>Procedure</u>

Before the first measurement the Edinburgh Handedness Inventory (Oldfield 1971) is filled in; only right handed participants are included. This questionnaire consists of 10 Items and it takes 1 minute to fill in.

Before each training and test session, a standard protocol is conducted in order to fit the simulator. The simulator is fitted with help of Otto Bock PAULA®. The electrodes within the simulator have to be placed on the optimal locations. The settings need to be tailored to each individual in order to record the myo-electric signal properly. The sensitivity of the electrodes will be adjusted for each participant on each day, so that all participants can just reach the myo-electric threshold of 1.5 V (high signal) and hold it for 2 seconds. The maximum speed of the hand is set to the default setting of 6 (range = 1-6). After the simulator is installed, the participant will be seated at a table and the session will start.

Prior to each task, during the training as well as the tests, the experimenter gives the participant instructions to execute the task. The participants are told to sit comfortably at a table, with their arms resting on the table. They start each task with the prosthetic hand closed. The participants are instructed to execute the tasks as rapidly as possible.

Pretest
During all tests we will use the Optotrak system to measure the movements of the arm and prosthesis. Two markers placed on the finger tips of the prosthetic arm; one on the thumb and one on the index finger.
At the first day, the test tasks are administered to determine the level of skills of the participants of the experimental and control groups. This test is performed with the test hand.

Training sessions
For the experiment different intervals between the training sessions are used. The training program in which the spacing will be varied is based on the findings of a previous experiment ; the most effective training of this experiment will be chosen to use for the second
Again, the most effective spacing is used in the study on patients.

Posttest and retention test
All participants perform a posttest, equal to the pretest. On day 10 and two weeks after the posttest these participants execute a retention test. For both tests the participants again perform the test tasks, in order to determine the improvement of skills and compare the different groups. The tasks are once more presented in a randomized order and executed with the test hand.

8.4 Withdrawal of individual subjects
Participants can leave the study at any time for any reason if they wish to do so without any consequences. The investigator can decide to withdraw a participant from the study for urgent medical reasons. Since the experiments are safe, we do not expect any urgent medical occasions.

8.4.1 Specific criteria for withdrawal (if applicable)
Not applicable

8.5 Replacement of individual subjects after withdrawal
If a participant withdraws from the study, another participant will be asked to join the study, preferably of the same sex.

8.6 Follow-up of subjects withdrawn from treatment
Not applicable

8.7 Premature termination of the study
Not applicable

9. SAFETY REPORTING

9.1 Section 10 WMO event
In accordance to section 10, subsection 1, of the WMO, the investigator will inform the participants and the reviewing accredited METC if anything occurs, on the basis of which it appears that the disadvantages of participation may be significantly greater than was foreseen in the research proposal. The study will be suspended pending further review by the accredited METC, except insofar as suspension would jeopardise the participants' health. The investigator will take care that all participants are kept informed.

9.2 AEs, SAEs and SUSARs

9.2.1 Adverse events (AEs)

Adverse events are defined as any undesirable experience occurring to a subject during the study, whether or not considered related to the experimental intervention (e.g. prosthetic simulator training or testing). All adverse events reported spontaneously by the subject or observed by the investigator or his staff will be recorded.

9.2.2 Serious adverse events (SAEs)

A serious adverse event is any untoward medical occurrence or effect that at any dose: results in death;

- is life threatening (at the time of the event);
- requires hospitalisation or prolongation of existing inpatients' hospitalisation;
- results in persistent or significant disability or incapacity;
- is a congenital anomaly or birth defect;

Any other important medical event that may not result in death, be life threatening, or require hospitalization, may be considered a serious adverse experience when, based upon appropriate medical judgement, the event may jeopardize the subject or may require an intervention to prevent one of the outcomes listed above.
The investigator is responsible for reporting all SAE's to the sponsor. This is independent of the centre where the research takes place. The sponsor will report the SAEs through the web portal ToetsingOnline to the accredited METC that approved the protocol, within 15 days after the sponsor has first knowledge of the serious adverse reactions.

SAEs that result in death or are life threatening should be reported expedited. The expedited reporting will occur not later than 7 days after the responsible investigator has first knowledge of the adverse reaction. This is for a preliminary report with another 8 days for completion of the report.

9.3 Annual safety report
Not applicable

9.4 Follow-up of adverse events
Not applicable

9.5 Data Safety Monitoring Board (DSMB)/Safety Committee

Not applicable

10. STATISTICAL ANALYSIS

10.1 Primary study parameter(s)

Deviation of grip force control,
Deviation of straight reaching path,
Length of plateau phase in grasping,
Movement time.

10.2 Secondary study parameter(s)

Age
Gender

10.3 Other study parameters

Not applicable

10.4 Analysis (if applicable)

In the experiments all measurements (deviation of grip force control, deviation of straight reaching path, length of plateau phase in grasping and movement time) are subjected to a repeated-measures ANOVA with test (pre-test, post-test and retention test(s)) as within-subject factor and dominance (preferred, non-preferred) and group (two experimental groups, two control groups) as between-subject factors.
When a Mauchly test indicates that sphericity is violated, the degrees of freedom are adjusted with the Greenhouse-Geisser correction. In all analyses, a significant criterion of α less than or equal to 0.05 is used, and post hoc tests on main effects use Bonferroni adjustment.

11. ETHICAL CONSIDERATIONS

11.1 Regulation statement

The study will be conducted according to the principles of the Declaration of Helsinki (59, October 2008) and in accordance with the Medical Research Involving Human Subjects Act.

11.2 Recruitment and consent

The able-bodied participants will be recruited by advertisement on publication boards of different faculties of the University of Groningen, and information presented in course lectures of Human Movement Sciences and Medicine by the investigator.

The participants will receive an information letter, with written information about the experiment, after they have shown interest in participating in the experiment. Participants will get between 1 and 8 weeks to decide whether they would like to join the study. For each potential participant there is the possibility to consult the researcher or an independent physician for any further information, this is also mentioned in the letter. After participants have signed in, they will sign an informed consent before the start of the experiment and it will be explained to them that they can stop with the experiment at any time without giving a reason. This can be done by telling the researchers.

11.3 Objection by minors or incapacitated subjects (if applicable)

Not applicable

11.4 Benefits and risks assessment, group relatedness

The participants have to learn to use a simulator during training sessions and will be tested on their abilities. All training sessions are done with non-injured hands and the measurements are non-invasive. Therefore, the risks associated with participation can be considered negligible and the burden can be considered minimal.

11.5 Compensation for injury

Because participation in the experiment is without risks, the judging committee, the METc UMCG has granted a release from compulsory insurance, as referred to in section 4 paragraph 1 of the 'Besluit verplichte verzekering bij medisch-wetenschappelijk onderzoek met mensen'.

11.6 Incentives (if applicable)

Not applicable

12. ADMINISTRATIVE ASPECTS, MONITORING AND PUBLICATION

12.1 Handling and storage of data and documents

The data is handled confidentially and coded for each participant; each participant will be given a number from 1 to 100. The investigator will keep the data for the duration of the project. The handling of personal data is complied with the Dutch Personal Data Protection Act (De Wet Bescherming Persoonsgegevens, Wbp).

12.2 Monitoring and Quality Assurance

Monitoring of the conduct of the study takes place by R.M. Bongers. The aim is to verify the rights and well-being of the participants, to check if the reported information is correctly derived from the original data and if the execution of the experiment in consensus is with the protocol, with good clinical practice and relevant laws. The inclusion of participants, the possible advents, the execution of the study and the progress of the study are monitored. The monitoring takes place at least once a week and in the lab where the experiments are conducted.

12.3 Amendments

Amendments are changes made to the research after a favourable opinion by the accredited METC has been given. All amendments will be notified to the METC that gave a favourable opinion.

The amendments will be implemented after the METc gives a positive judgement.

Non-substantial amendments will not be notified to the accredited METC and the competent authority, but will be recorded and filed by the sponsor.

12.4 Annual progress report

The investigator will submit a summary of the progress of the trial to the accredited METC after a year. Information will be provided on the date of inclusion of the first subject, numbers of subjects included and numbers of subjects that have completed the trial, serious adverse events/ serious adverse reactions, other problems and amendments.

12.5 End of study report

The investigator will notify the accredited METC of the end of the study within a period of 8 weeks. The end of the study is defined as the last patient's last visit.

In case the study is ended prematurely, the investigator will notify the accredited METC, including the reasons for the premature termination.

Within one year after the end of the study, the investigator/sponsor will submit a final study report with the results of the study, including any publications/abstracts of the study, to the accredited METC.

12.6 Public disclosure and publication policy

The results of this study will be unreservedly published in a peer reviewed scientific journal.

13. STRUCTURED RISK ANALYSIS

In this study a medical device, e.g. the prosthetic simulator, will be used.

13.1 Potential issues of concern

In this study we will use a medical device, namely the prosthetic simulator. We expect that this study has no risk of potential issues of concern for the participants. The used prosthetic simulator is an approved medical device (approval is added). Furthermore, the device is used before in two experiments of our study group (NL26993.042.09 en NL35268.042.11).

13.2 Synthesis

Not applicable

REFERENCES

ATKINS, D., 1992. Adult Upper Limb Prosthetic Training. In: H.K. BOWKER and J.W. MICHAEL, eds, *Atlas of Limb Prosthetics: Surgical, Prosthetic, and Rehabilitation Principles.* 2 edn. Rosemont, I.L., .

BENSMAIL, D., SARFELD, A.S., FINK, G.R. and NOWAK, D.A., 2010. Intermanual transfer of sensorimotor memory for grip force when lifting objects: the role of wrist angulation. *Clinical neurophysiology : official journal of the International Federation of Clinical Neurophysiology,* **121**(3), pp. 402-407.

CHANG, E.C., FLANAGAN, J.R. and GOODALE, M.A., 2008. The intermanual transfer of anticipatory force control in precision grip lifting is not influenced by the perception of weight. *Experimental brain research.Experimentelle Hirnforschung.Experimentation cerebrale,* **185**(2), pp. 319-329.

CHRISTOU, E.A. and RODRIGUEZ, T.M., 2008. Time but not force is transferred between ipsilateral upper and lower limbs. *Journal of motor behavior,* **40**(3), pp. 186-189.

DAKPA, R. and HEGER, H., 1997. Prosthetic management and training of adult upper limb amputees. *Current Orthopaedics,* **11**, pp. 193-202.

DONOVAN, J.J. and RADOSEVICH, D.J., 1999. A Meta-Analytic Review of the Distribution of Practice Effect: Now you see it, now you don't. *Journal of Applied Psychology,* **84**(5), pp. 795-805.

FARTHING, J.P., KRENTZ, J.R., MAGNUS, C.R., BARSS, T.S., LANOVAZ, J.L., CUMMINE, J., ESOPENKO, C., SARTY, G.E. and BOROWSKY, R., 2011. Changes in functional magnetic resonance imaging cortical activation with cross education to an immobilized limb. *Medicine and science in sports and exercise,* **43**(8), pp. 1394-1405.

GAINE, W.J., SMART, C. and BRANSBY-ZACHARY, M., 1997. Upper limb traumatic amputees. Review of prosthetic use. *Journal of hand surgery (Edinburgh, Scotland),* **22**(1), pp. 73-76.

GOEDERT, K.M. and MILLER, J., 2008. Spacing practice sessions across days earlier rather than later in training improves performance of a visuomotor skill. *Experimental brain research.Experimentelle Hirnforschung.Experimentation cerebrale,* **189**(2), pp. 189-197.

HICKS, R.E., GUALTIERI, C.T. and SCHOEDER, S.R., 1983. Cognitive and motor components of bilateral transfer. *American Journal of Psychology,* **96**(2), pp. 223-228.

HUSSAIN, Z., SEKULER, A.B. and BENNETT, P.J., 2009. How much practice is needed to produce perceptual learning? *Vision research,* **49**(21), pp. 2624-2634.

KARNI, A., MEYER, G., REY-HIPOLITO, C., JEZZARD, P., ADAMS, M.M., TURNER, R. and UNGERLEIDER, L.G., 1998. The acquisition of skilled motor performance: fast and slow experience-driven changes in primary motor cortex. *Proceedings of the National Academy of Sciences of the United States of America,* **95**(3), pp. 861-868.

KORNMEIER, J. and SOSIC-VASIC, Z., 2012. Parallels between spacing effects during behavioral and cellular learning. *Frontiers in human neuroscience,* **6**, pp. 203.

KUMAR, S. and MANDAL, M.K., 2005. Bilateral transfer of skill in left- and right-handers. *Laterality,* **10**(4), pp. 337-344.

LEE, M. and CARROLL, T.J., 2007. Cross education: possible mechanisms for the contralateral effects of unilateral resistance training. *Sports medicine (Auckland, N.Z.),* **37**(1), pp. 1-14.

LEE, M., HINDER, M.R., GANDEVIA, S.C. and CARROLL, T.J., 2010. The ipsilateral motor cortex contributes to cross-limb transfer of performance gains after ballistic motor practice. *Journal of Physiology,* **558**(1), pp. 201-212.

LIGHT, C.M., CHAPPELL, P.H. and KYBERD, P.J., 2002. Establishing a standardized clinical assessment tool of pathologic and prosthetic hand function: normative data, reliability, and validity. *Archives of Physical Medicine and Rehabilitation,* **83**(6), pp. 776-783.

MALONE, J.M., FLEMING, L.L., ROBERSON, J., WHITESIDES, T.E.,Jr, LEAL, J.M., POOLE, J.U. and GRODIN, R.S., 1984. Immediate, early, and late postsurgical management of upper-limb amputation. *Journal of rehabilitation research and development,* **21**(1), pp. 33-41.

MIER, H.I. and PETERSEN, S.E., 2006. Intermanual transfer effects in sequential tactuomotor learning: Evidence for effector independent coding. *Neuropsychologia,* **44**(1), pp. 939-949.

OLDFIELD, R.C., 1971. The assessment and analysis of handedness: the Edinburgh inventory. *Neuropsychologia,* **9**(1), pp. 97-113.

PARK, J.H. and SHEA, C.H., 2002. Effector independence. *Journal of motor behavior,* **34**(3), pp. 253-270.

PEREIRA, E.A., RAJA, K. and GANGAVALLI, R., 2011. Effect of training on interlimb transfer of dexterity skills in healthy adults. *American Journal of Physical Medicine & Rehabilitation / Association of Academic Physiatrists,* **90**(1), pp. 25-34.

ROMKEMA, S., BONGERS, R.M. and VAN DER SLUIS, C.K., 2013. Intermanual transfer in training with an upper-limb myoelectric prosthesis simulator: a mechanistic, randomized, pretest-posttest study. *Physical Therapy,* **93**(1), pp. 22-31.

SCHMIDT, R.A. and LEE, T.D., eds, 2005. *Motor Control and learning: a behavioral emphasis.* 4 edn. Champaign, United States of America: Human Kinetics.

SHEA, C.H., LAI, Q., BLACK, C. and PARK, J.H., 2000. Spacing practice sessions across days benefits the learning of motor skills. *Human Movement Science,* **19**, pp. 737-760.

SIENGSUKON, C.F. and BOYD, L.A., 2009. Does sleep promote motor learning? Implications for Physical Rehabilitation. *Physical Therapy,* **89**(4), pp. 370-383.

TEIXEIRA, L.A., 2000. Timing and force components in bilateral transfer of learning. *Brain and cognition,* **44**(3), pp. 455-469.

VAN LUNTEREN, A., VAN LUNTEREN-GERRITSEN, G.H., STASSEN, H.G. and ZUITHOFF, M.J., 1983. A field evaluation of arm prostheses for unilateral amputees. *Prosthetics and orthotics international,* **7**(3), pp. 141-151.

WEEKS, D.L., WALLACE, S.A. and ANDERSON, D.I., 2003. Training with an upper-limb prosthetic simulator to enhance transfer of skill across limbs. *Archives of Physical Medicine and Rehabilitation,* **84**(3), pp. 437-443.
